# Advancing precision cardio-oncology: multimodal surveillance and stratified management of immunotherapy-induced cardiovascular toxicity

**DOI:** 10.3389/fcvm.2026.1891563

**Published:** 2026-09-18

**Authors:** Bihan Sun, Song Guo

**Affiliations:** Department of Cardiac Surgery, The First Hospital of China Medical University, Shenyang, China

**Keywords:** cardio-oncology, cardiovascular toxicity, CAR-T cell therapy, cytokine release syndrome (CRS), immune checkpoint inhibitors (ICIs), myocarditis

## Abstract

The advent of immune checkpoint inhibitors (ICIs) and chimeric antigen receptor T-cell (CAR-T) therapies has revolutionized oncology but is increasingly associated with severe cardiovascular immune-related adverse events (irAEs). This narrative review comprehensively examines the epidemiology, pathophysiologic mechanisms, biomarkers, advanced imaging, and management strategies for immunotherapy-associated cardiovascular toxicities. ICI-associated myocarditis is a rare but potentially fatal complication occurring early in therapy, particularly with combination regimens, while the broader cardiovascular spectrum also encompasses pericarditis, arrhythmias, heart failure, and accelerated atherosclerosis. Mechanistically, preclinical data suggest that ICI-associated myocarditis is hypothesized to be mediated by autoreactive CD8+ T-cell cross-reactivity against shared cardiac antigens, macrophage expansion, and inflammatory cytokine signaling, whereas CAR-T cardiovascular toxicity appears to contribute primarily secondary to systemic cytokine release syndrome (CRS). For early detection and risk stratification, high-sensitivity cardiac troponin remains the foundational biomarker, complemented by advanced multimodal imaging such as global longitudinal strain (GLS) echocardiography, cardiac magnetic resonance (CMR), and emerging molecular positron emission tomography (PET). High-dose systemic corticosteroids remain the first-line intervention for symptomatic ICI-associated myocarditis, with targeted therapies like abatacept and ruxolitinib showing promise for steroid-refractory cases. Ultimately, proactive risk factor assessment and multidisciplinary cardio-oncology surveillance are imperative to mitigate acute toxicities and long-term atherosclerotic risks, ensuring optimal patient outcomes.

## 1 Introduction

The advent of immune checkpoint inhibitors (ICIs) and chimeric antigen receptor T-cell (CAR-T) therapies has revolutionized the treatment landscape for a wide range of hematologic malignancies and solid tumors (1–3). These immunotherapies, which activate the host immune system to target cancer cells, have provided significant survival benefits for patients who previously had poor prognoses (4, 5). However, this potent immune activation is not entirely specific and frequently leads to a spectrum of immune-related adverse events (irAEs) that can affect virtually any organ, including the cardiovascular system (6–8). Although cardiovascular irAEs are relatively rare, they carry substantial morbidity and mortality, posing a formidable clinical challenge and catalyzing the emergence of cardio-oncology as a distinct subspecialty (9–11).

Among ICI-associated cardiovascular toxicities, myocarditis is the most recognized and life-threatening manifestation (12, 13). Although fatality rates have declined from the nearly 50% reported in early series due to increased clinical awareness and improved management, ICI-associated myocarditis remains a major cause of treatment interruption and patient death (11, 14, 15). Beyond myocarditis, ICIs are associated with a broad spectrum of other cardiovascular complications (16–18). Emerging evidence suggests that prolonged ICI use may exacerbate inflammation within atherosclerotic plaques, thereby increasing the long-term risk of myocardial infarction and ischemic stroke (7, 12, 19).

In contrast to ICIs, cardiovascular toxicity associated with CAR-T cell therapy is primarily secondary to its most common complication, cytokine release syndrome (CRS) (20, 21). Notably, patients with pre-existing cardiovascular conditions such as advanced age, prior atrial arrhythmia, hypertension, heart failure, or coronary artery disease are at significantly higher risk for CAR-T-associated cardiovascular toxicity (22).

Understanding the pathophysiologic mechanisms underlying these immunotherapy-related cardiovascular toxicities is critical for developing effective risk stratification, monitoring, prevention, and treatment strategies (1, 23). The pathogenesis of ICI-associated myocarditis involves T-cell recognition and attack of cardiomyocytes expressing shared antigens, cytokine-mediated injury, and autoantibody involvement (15, 24, 25). In contrast, CAR-T-associated cardiac injury is thought to be driven by CRS-mediated systemic inflammation, capillary leak syndrome, and myocardial depression (21, 26). Recent advances in novel biomarkers (e.g., high-sensitivity troponin, inflammatory cytokines) and advanced imaging modalities [e.g., cardiac magnetic resonance (CMR)] have provided new tools for early detection and risk stratification (27–29). Furthermore, a deeper understanding of risk factors, such as combination immunotherapy, pre-existing autoimmune disease, and prior cardiovascular history, has laid the groundwork for individualized prevention strategies (30, 31). This narrative review aims to comprehensively discuss the epidemiology, pathophysiologic mechanisms, biomarkers, imaging assessment, risk factors and prevention strategies, as well as current management approaches and emerging therapeutic options for immunotherapy-associated cardiovascular toxicity, providing a comprehensive reference for clinical practice and future research. While encompassing the broader spectrum of immunotherapy-induced cardiovascular complications, this review is deliberately centered on ICI-associated myocarditis—the most fulminant, lethal, and extensively studied presentation—while providing a synthesized overview of emerging non-myocarditis toxicities.

## 2 Epidemiology and clinical Spectrum of immunotherapy-associated cardiovascular toxicity

The recognition of cardiovascular toxicity as a significant adverse effect of ICIs has evolved substantially since the initial reports of fulminant myocarditis. Early estimates from clinical trials suggested a relatively low incidence of ICI-associated myocarditis, ranging from 0.04% to 1.14% (13, 32, 33). However, as clinical experience has expanded and awareness has grown, it has become increasingly clear that the true incidence is likely higher than initially appreciated, particularly when subclinical or mild presentations are considered (32, 34). A large systematic review and meta-analysis encompassing 83,315 participants across 589 clinical trials reported an overall incidence of cardiovascular adverse events (CVAEs) of 0.80% for anti-programmed cell death protein 1 (PD-1)/programmed death-ligand 1 (PD-L1) monotherapy and 1.07% for anti-cytotoxic T-lymphocyte-associated antigen 4 (CTLA-4) monotherapy (14). The incidence of myocarditis was significantly higher with dual ICI therapy (anti-CTLA-4 combined with anti-PD-1/PD-L1), a finding consistently replicated across multiple studies (14, 32, 35). Prospective multimodal surveillance studies reveal that early major adverse cardiovascular events (MACE) occur at a significantly higher rate than previously recognized in retrospective analyses (34). These data suggest that when systematic detection strategies are applied, the burden of ICI-associated cardiovascular toxicity may be considerably higher than previously reported in retrospective analyses (34, 36).

ICI-associated myocarditis typically presents early, with a median onset of approximately 4 weeks following treatment initiation across multiple registries and pharmacovigilance studies (32, 37, 38). This early temporal pattern distinguishes ICI-associated myocarditis from many other irAEs and underscores the need for heightened vigilance during the initial weeks of therapy (16, 32, 39). The clinical presentation of ICI-associated myocarditis is highly variable, ranging from asymptomatic cardiac biomarker elevation to fulminant heart failure, cardiogenic shock, and sudden cardiac death (12, 32, 39). This observation highlights the limitations of relying solely on ejection fraction for risk assessment and emphasizes the importance of comprehensive evaluation including biomarkers and advanced imaging (32, 40). The spectrum of disease has broadened over time, with contemporary reports describing smoldering, nonsevere, and severe or fulminant forms of myocarditis (12). Fatality rates, while still substantial, have declined from initial estimates approaching 50% to more recent reports of 25%–40%, likely reflecting improved recognition and earlier intervention (11, 12, 14, 15).

Beyond myocarditis, ICI therapy is associated with a diverse array of other cardiovascular toxicities. Pericarditis and pericardial effusions, which can range from asymptomatic to life-threatening cardiac tamponade, have been increasingly recognized (2, 12, 17). In a large retrospective cohort study of 2,165 patients treated with ICIs, dual ICI therapy was associated with a 2.0-fold increased risk of myo/pericarditis, and the occurrence of myo/pericarditis was associated with a 2.9-fold increased risk of mortality (29). Arrhythmias, including atrial fibrillation, supraventricular tachycardias, ventricular arrhythmias, and high-degree heart block, are common complications that can occur in the setting of myocarditis, ischemia, or systemic inflammation (12, 17, 41). Takotsubo syndrome, or stress-induced cardiomyopathy, has also been reported as a distinct entity associated with ICI therapy, presenting with transient left ventricular systolic dysfunction mimicking acute myocardial infarction (16, 17, 41). Non-inflammatory heart failure, characterized by cardiac dysfunction without evidence of myocardial inflammation, has been observed in ICI-treated patients and may represent a distinct pathophysiological entity (12, 17, 18).

Accelerated atherosclerosis and its clinical consequences represent an emerging and potentially more prevalent long-term cardiovascular complication of ICI therapy (2, 16–18). Preclinical and clinical evidence suggests that ICIs may promote plaque inflammation and progression of atherosclerosis, leading to an increased risk of myocardial infarction and stroke (12, 18, 42). While acute ischemic events during active treatment are not frequent, the long-term implications of ICI-associated atherosclerotic progression are only beginning to be understood, and this may represent a significant contributor to cardiovascular morbidity in cancer survivors (12, 17, 18). A meta-analysis of randomized controlled trials found that ICI use was associated with a higher risk of myocardial infarction (OR, 1.83) compared with non-ICI therapy (35). Vasculitis, including giant cell arteritis and other forms, has also been reported as a rare but serious immune-related cardiovascular adverse event (12, 18, 43).

Several risk factors for ICI-associated cardiovascular toxicity have been identified. Combination ICI therapy (anti-CTLA-4 plus anti-PD-1/PD-L1) is the most consistently reported and potent risk factor for developing myocarditis (14, 31, 32, 39). In a meta-analysis of observational studies, combination ICIs demonstrated a 3.07-fold higher risk of myocarditis compared with single ICI use (35). Preexisting cardiovascular disease, including coronary artery disease and heart failure, has been associated with an increased risk of ICI-associated myocarditis and adverse outcomes (31, 40, 44). Diabetes mellitus was also more common in patients who developed myocarditis in the initial multicenter registry (32). Other reported risk factors include older age, male sex, prior arrhythmia, and the presence of lung cancer or central nervous system malignancies (29, 31, 40). A pharmacovigilance study identified body weight ≥75 kg and the use of nivolumab and ipilimumab as potential significant risk factors for noninfectious myocarditis/pericarditis (38).

The clinical spectrum of ICI-associated cardiovascular toxicity continues to expand as experience with these agents grows. Myocarditis remains the most concerning and most studied toxicity due to its high mortality rate, but the recognition of other cardiovascular complications such as pericarditis, arrhythmias, Takotsubo syndrome, non-inflammatory heart failure, and accelerated atherosclerosis has broadened the scope of cardio-oncology care required for these patients (2, 3, 12, 18). The heterogeneity of clinical presentations, ranging from asymptomatic biomarker elevation to catastrophic cardiac events, poses significant diagnostic challenges (13, 32, 39). ICI-associated myocarditis frequently occurs in the absence of other irAEs, with 54% of patients having no other irAEs in the initial multicenter registry (32). This lack of concurrent systemic immune activation can delay diagnostic consideration and underscores the need for a high index of suspicion (16, 25, 32). Furthermore, ICI-associated myocarditis often coexists with myositis and myasthenia gravis, forming an overlap syndrome that is associated with particularly fulminant presentations and high early mortality (37, 43, 44). In one series, 66.7% of patients with ICI-associated myocarditis also had myasthenia gravis-like overlap syndrome and 50% had myositis or rhabdomyolysis (44). A multicenter study identified concomitant ICI-myositis as an independent predictor of cardiovascular toxicity-related death (37). These overlap syndromes require careful multidisciplinary management involving cardiologists, neurologists, and oncologists (43, 44).

The prognostic implications of ICI-associated cardiovascular toxicity are substantial. In a large international registry of 757 patients hospitalized with ICI-associated myocarditis, 35% presented with a reduced left ventricular ejection fraction (LVEF) (<50%), which was marginally associated with 30-day all-cause mortality (adjusted HR, 1.50) (40). Predictors of reduced LVEF included prior chest radiation, pre-existing heart failure, dyspnea at presentation, and a longer interval from ICI initiation to myocarditis onset (≥40 days) (40). In contrast, myositis symptoms were associated with preserved LVEF (40). Elevated cardiac biomarkers, particularly troponin, have been consistently associated with adverse outcomes. In the initial registry, a troponin T level ≥1.5 ng/mL was associated with a 4-fold increased risk of MACE (32). A multicenter study identified cardiac troponin I (cTnI) ≥ 50 times the upper reference limit and LVEF < 50% as strong predictors of shortened survival, with these factors influencing early but not long-term mortality beyond 60 days (37). Creatine kinase (CK)-MB and neutrophil-to-lymphocyte ratio were identified as independent risk factors for the development of ICI-associated myocarditis and severe myocarditis, respectively (45). Despite the high short-term mortality, long-term prognosis among survivors appears comparable to patients without myocarditis, suggesting that early aggressive intervention may mitigate the risk of chronic cardiac dysfunction (37) (Table 1).

**Table 1:** Epidemiological profiles and clinical spectrums of immunotherapy-associated cardiovascular toxicities.

Therapy Type	Core Manifestations	Incidence/Risk Profile	Median Onset	Distinctive Clinical Features
ICI Monotherapy	Myocarditis, pericarditis, non-inflammatory heart failure, accelerated atherosclerosis	0.80% (anti-PD-1/PD-L1) to 1.07% (anti-CTLA-4)	28–34 days	Highly variable presentation ranging from asymptomatic biomarker elevation to fulminant heart failure; frequently presents without concurrent non-cardiac irAEs
ICI Combination	Fulminant myocarditis, severe arrhythmias, cardiogenic shock	∼3.07-fold higher risk of myocarditis compared to single ICI use	∼28 days	High mortality rates of 25%–40%; frequently forms an overlap syndrome with myositis and myasthenia gravis
CAR-T Cell Therapy	Hemodynamic instability, arrhythmias, left ventricular systolic dysfunction	19.68% overall (arrhythmias: 7.70%, heart failure: 5.73%, troponin elevation: up to 23.61%)	Acute phase (post-infusion)	Toxicity is primarily secondary to systemic CRS; hypothesized to be driven by cytokine-mediated myocardial stunning rather than direct infiltration

## 3 Pathophysiological mechanisms of ICI- and CAR-T cell-related cardiovascular toxicity

The pathophysiological basis of immunotherapy-associated cardiovascular toxicity is fundamentally rooted in the unintended activation of the immune system against self-antigens, driven by the very mechanisms that confer therapeutic efficacy against malignancies. For ICIs, the central paradigm involves the disruption of immune tolerance checkpoints—primarily CTLA-4 and PD-1/PD-L1 pathways—which are physiologically critical for maintaining peripheral self-tolerance and preventing autoimmunity (1, 23, 46). Unlike conventional cardiotoxic agents that directly injure cardiomyocytes, ICI-associated cardiovascular toxicity results from an exaggerated adaptive immune response, most notably T cell-mediated cross-reactivity against cardiac tissues (47–49).

A proposed foundational mechanism in ICI-associated myocarditis is molecular mimicry, wherein T cells activated against tumor-specific antigens are hypothesized to also recognize shared epitopes expressed on cardiac tissue (48, 49). This concept is supported by the identification of cardiac myosin-specific autoimmune T cells as critical drivers of disease. Specifically, mouse models demonstrate that pre-existing cardiac myosin-specific autoimmune T cells are unleashed upon immune checkpoint blockade, driving myocardial infiltration and injury (50). Further evidence demonstrates that the combination of CTLA-4 and PD-1 blockade uniquely drives robust expansion of antigen-specific CD8+ T cells, leading to myocardial inflammation and lethal arrhythmias in mice expressing a cardiomyocyte-restricted antigen (51).

The cellular landscape of ICI-associated myocarditis is dominated by T lymphocytes, but the specific subsets and their relative contributions have been increasingly refined. Preclinical and clinical data indicate that CTLA-4 inhibition preferentially results in giant cell myocarditis with a predominately CD4+ T cell infiltrate, whereas PD-1 inhibition leads to lymphocytic myocarditis with a predominately CD8+ T cell infiltrate (52). This distinction highlights that different checkpoint targets may precipitate distinct pathological patterns. However, the overwhelming consensus from both mouse models and human studies implicates effector CD8+ T cells as the primary mediators of myocardial injury in the most common and fulminant forms of ICI-associated myocarditis (46, 53, 54). Preclinical data suggest that these autoreactive CD8+ T cells may directly damage cardiomyocytes, and their expansion appears to be a hallmark of the disease.

Beyond the direct cytotoxic role of T cells, a growing body of evidence highlights the critical involvement of macrophages and the inflammatory milieu they create. Single-cell RNA-sequencing studies in a murine model of ICI-associated myocarditis (Ctla4+/‒Pdcd1^‒/‒^ mice) have revealed marked expansion of CCR2+ monocyte-derived macrophages alongside CD8+ T cells (55). In silico analyses suggested interactions between T cells and these Cxcl9 + Cxcl10+ macrophages via interferon-*γ* (IFN-*γ*) and CXCR3 signaling pathways, and depleting CD8+ T cells, macrophages, or blocking IFN-*γ* signaling blunted the expansion of these macrophages and attenuated myocarditis (55). These data establish a feed-forward loop where T cell-derived IFN-*γ* drives the recruitment and activation of inflammatory macrophages, which in turn amplify tissue damage.

The importance of macrophages is further underscored by clinicopathological studies. In a cohort of patients with suspected ICI-associated myocarditis, traditional Dallas criteria identified only a minority of endomyocardial biopsy specimens, but supplemental immunohistochemical staining for CD68+ macrophages identified additional cases with pathological features of myocardial inflammation (56). Macrophage abundance positively correlated with serum troponin I and N-terminal pro-B-type natriuretic peptide (NT-proBNP) concentrations, and inclusion of CD68 immunohistochemistry could have changed the diagnostic certainty in a substantial proportion of cases (56). This suggests that macrophage infiltration is a consistent and quantifiable feature of ICI-associated myocarditis that may be underappreciated by conventional histological assessment.

Cytokine signaling constitutes a central mechanism driving both the recruitment of immune cells and the resultant cardiac dysfunction. A variety of inflammatory cytokines have been implicated, including tumor necrosis factor-alpha (TNF-α), IFN-*γ*, interleukin-1β (IL-1β), interleukin-6 (IL-6), and interleukin-17A (IL-17A) (57, 58). The role of IL-17A has been particularly well-characterized. In a murine model, anti-PD-1 treatment led to cardiac dysfunction and left ventricular dilation, with enhanced thymic inflammatory signaling where Il17a increased most prominently (59). Pharmacological inhibition of IL-17A prevented anti-PD-1-induced cardiac dysfunction, demonstrating that this cytokine is not merely a bystander but a critical driver of functional impairment (59). The importance of the TNF axis has also been mechanistically defined. Using a mouse model with cardiomyocyte-restricted antigen expression, it was shown that myocardial injury was independent of perforin-mediated cytotoxicity but critically depended on T cell-derived TNF, which promoted myeloid recruitment, cytokine production, and arrhythmogenesis (51). Genetic ablation of CD8 T cell-derived TNF or blockade of TNF receptor 2 (TNFR2) prevented cardiovascular toxicity while preserving antitumor efficacy, identifying a specific signaling circuit downstream of autoreactive CD8 T cells as a central mechanism and a potential therapeutic target (51).

Combinatorial immune checkpoint blockade, a well-documented risk factor for myocarditis, amplifies these inflammatory pathways. *In vitro* studies using human cardiomyocytes co-cultured with peripheral blood lymphocytes demonstrated that combinations of PD-L1/CTLA-4 or PD-1/LAG-3 blocking agents exert more potent cardiotoxic effects compared to monotherapies (60). These combinatorial regimens induced greater release of lactate dehydrogenase, troponin-T, B-type natriuretic peptide (BNP), and heart-type fatty acid binding protein (H-FABP), and significantly increased NLRP3 inflammasome expression along with IL-6 and IL-1β secretion (60). Mitochondrial dysfunction, evidenced by reduced ATP content and Ndufs1 levels, along with increased intracellular Ca2+ and HDAC4 levels, further highlights the multi-faceted cellular injury induced by combined checkpoint inhibition (60).

Sex differences in susceptibility to ICI-associated myocarditis have emerged as a clinically relevant dimension of pathophysiology, with female patients appearing to be preferentially affected. Preclinical mechanistic studies have implicated an endocrine-cardiac-immune pathway involving mesencephalic astrocyte-derived neurotrophic factor (MANF) and heat shock 70-kDa protein 5 (HSPA5) (61). Treatment with an estrogen receptor β-specific agonist attenuated ICI-associated cardiac effects, suggesting that estrogen-dependent expression of these protective factors is curtailed by ICI therapy, increasing vulnerability to immune injury (61).

The presence of pre-existing cardiovascular injury has been identified as a critical prerequisite for the development of lethal ICI-associated myocarditis in experimental models. In a hypertension mouse model, ICI combination therapy (anti-CTLA-4 plus anti-PD-1) led to 30% increased mortality, with myocarditis developing exclusively in the hypertensive group treated with ICIs, characterized by T cell infiltration and increased cardiac antigen autoantibodies (62). The control group treated with ICIs alone was unaffected, indicating that a pre-existing state of cardiac stress or damage is necessary to lower the threshold for immune-mediated attack (62). This concept is further supported by transcriptomic analyses comparing mouse strains with different susceptibility to ICI-associated cardiac dysfunction. In C57BL/6J mice, which developed cardiac dysfunction after anti-PD-1 treatment, differentially regulated genes related to cardiac structure, signaling, and inflammation were identified compared to the resistant BALB/c strain (59).

The role of the cardiac immune microenvironment extends to the concept of immune checkpoint molecule expression on cardiac cells themselves. PD-L1 is expressed on cardiomyocytes and endothelial cells and serves as a protective “immune brake” against T cell-mediated attack. ICI therapy removes this brake, leaving cardiac cells vulnerable to immune-mediated injury (63). This local loss of immune tolerance within the heart is a critical factor that distinguishes susceptible individuals from those who remain unaffected.

In contrast to ICI-associated cardiovascular toxicity, the pathophysiology of cardiovascular toxicity from CAR-T cell therapy is primarily driven by CRS, a systemic inflammatory response triggered by massive T cell activation and proliferation (2, 42, 64). The infusion of CAR-T cells leads to activation of both the engineered T cells and host immune cells, resulting in a cytokine storm characterized by markedly elevated levels of IL-6, IL-1, IFN-*γ*, and TNF-α (64, 65). This hyperinflammatory state has direct and profound effects on the cardiovascular system, including myocardial depression, capillary leak syndrome, and hemodynamic compromise (2, 42). The cardiovascular consequences of high-grade CRS include arrhythmias, myocardial infarction, and heart failure, with an incidence of cardiovascular events as high as 20% among patients who develop severe CRS (64). The mechanism of myocardial injury in this context is largely indirect, driven by cytokine-mediated myocardial stunning and systemic vasodilation rather than direct T cell infiltration of the heart, although myocardial edema and stress can occur (65, 66). Treatment with IL-6 inhibitors (e.g., tocilizumab) and corticosteroids is associated with improved outcomes, directly targeting the cytokine axis central to CRS pathophysiology (42) (Figure 1).

**Figure 1:**
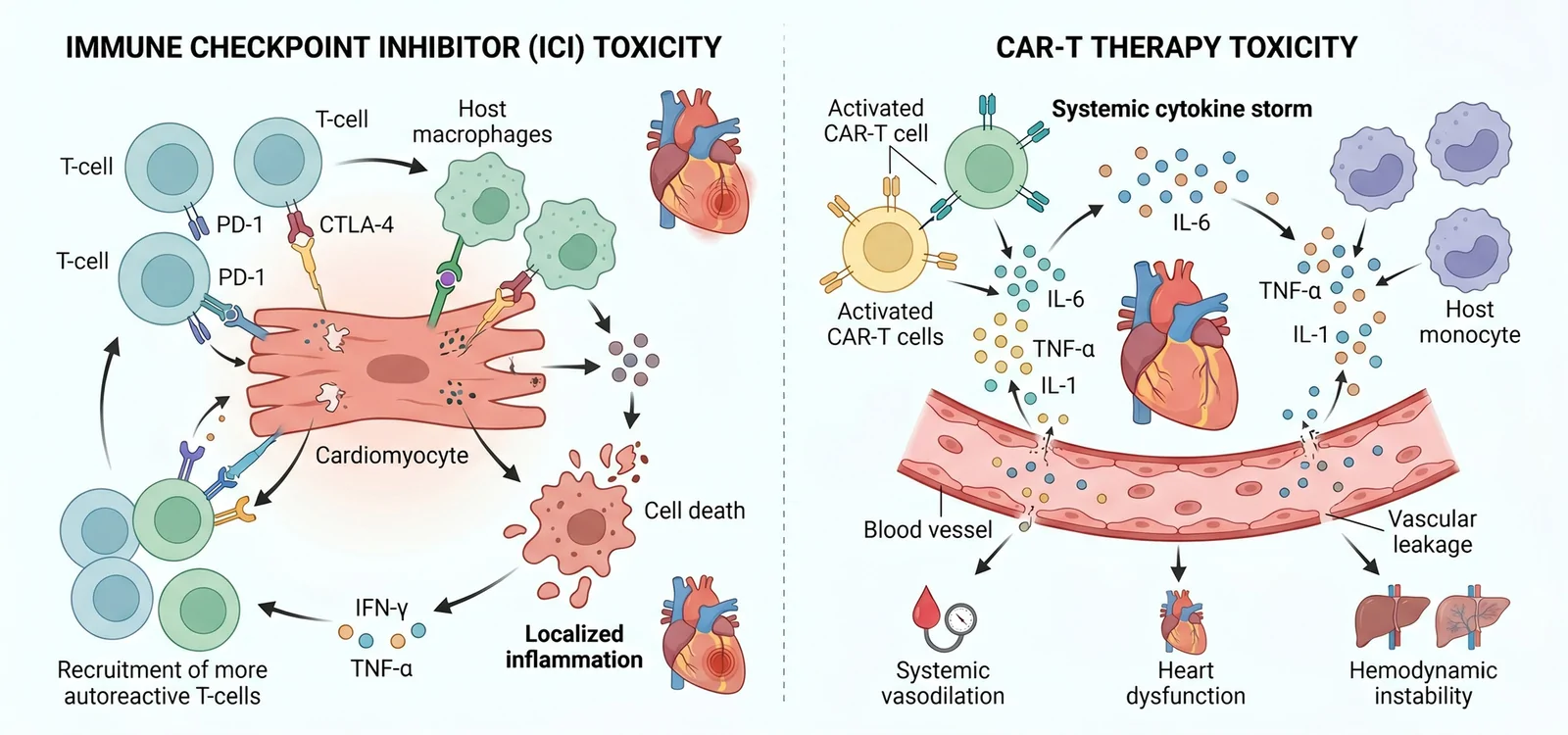
Proposed pathophysiological mechanisms of immunotherapy-induced cardiovascular toxicity.

The left panel illustrates the hypothesized mechanism of ICI-associated cardiovascular toxicity, which appears to be primarily driven by localized myocardial inflammation. Blockade of the PD-1 and CTLA-4 pathways leads to the hyperactivation of autoreactive T-cells and the subsequent recruitment of host macrophages. This cascade results in direct cardiomyocyte damage, cell death, and the local release of pro-inflammatory cytokines (IFN-*γ*, TNF-α). The right panel depicts the mechanism of CAR-T therapy-associated cardiovascular toxicity, which is predominantly secondary to a systemic cytokine storm. Activated CAR-T cells and host monocytes release massive amounts of circulating cytokines (IL-6, IL-1, TNF-α), triggering systemic vasodilation, vascular leakage, and hemodynamic instability, ultimately leading to global heart dysfunction without direct T-cell infiltration of the myocardium. (Created with BioRender.com).

## 4 Biomarkers for diagnosis, risk stratification, and prognosis

The identification and clinical application of biomarkers have become central to the management of immunotherapy-associated cardiovascular toxicity, particularly for ICI-associated myocarditis and CAR-T cell-associated cardiovascular events. Cardiac troponins, natriuretic peptides, and a range of inflammatory and hematologic markers have been investigated for their roles in early detection, risk stratification, and prognostication (28, 30, 67). The evolving evidence base supports the integration of these biomarkers into clinical algorithms, though significant challenges remain regarding assay standardization, optimal cutoffs, and the interpretation of asymptomatic elevations (68, 69). Crucially, to prevent the over-translation of preliminary data, it is imperative to explicitly distinguish these established standard-of-care assays from a growing pipeline of emerging investigational tools (such as novel cytokine panels, composite ratios, and proteomic signatures) which are not yet ready for routine clinical adoption.

Cardiac troponin (cTn) stands as the most extensively studied biomarker for ICI-associated myocarditis, serving as a cornerstone for both diagnosis and prognostication (67, 70). Elevated troponin levels are present in the vast majority of patients with confirmed ICI-associated myocarditis, and the magnitude of elevation correlates strongly with disease severity and adverse outcomes (27, 71, 72). In a comprehensive analysis of 748 patients from a multicenter international registry, incremental troponin elevation demonstrated a dose-response relationship with the primary composite outcome of severe arrhythmia, heart failure, respiratory muscle failure, or cardiomyotoxicity-related death (71, 72). This relationship underscores the utility of troponin not merely as a binary diagnostic tool but as a continuous variable for risk stratification. Derived from retrospective registry data, a prognostic risk score incorporating troponin magnitude, along with active thymoma, low QRS voltage, depressed LVEF, and cardiomuscular symptoms, demonstrated robust performance, with 30-day primary outcome incidence increasing from 4% in the lowest risk category to 81% in the highest (71, 72).

Important differences between troponin assays have emerged, with high-sensitivity cardiac troponin T (hs-cTnT) showing superior diagnostic sensitivity compared to cTnI in the setting of ICI-associated myocarditis (27). Furthermore, cTnT demonstrated stronger prognostic performance, with early significant elevations showing a strong positive correlation with the risk of major adverse cardiomyotoxic events (27). Notably, cTnT remained elevated in all patients within 72 h of the first major adverse event, whereas cTnI and CK were normal in 11% and 27% of patients, respectively, suggesting that cTnT may be more reliable for surveillance (27). The clinical significance of isolated troponin elevations in asymptomatic patients remains an area of active investigation (68, 73). A prospective study of 164 patients receiving ICI therapy found that significant hs-cTnT elevations occurred in 16% of patients, yet only 5% developed confirmed ICI-associated myocarditis, indicating that many troponin elevations are not associated with clinically apparent myocarditis (73). However, a retrospective cohort study of 428 patients who underwent routine serial cTnI monitoring demonstrated that troponin surveillance integrated with symptom-based triaging facilitated early intervention, with asymptomatic patients more often continuing immunotherapy while symptomatic patients underwent more intensive diagnostics and received immunosuppression (74). The cumulative incidence of MACE at 1.5 years following cTnI elevation was 19%, with symptomatic patients at significantly higher risk for both events and all-cause mortality (74). These findings support a proposed risk-based approach wherein troponin screening triggers further evaluation based on clinical context rather than leading to automatic treatment decisions, though the universal application of routine serial surveillance remains an expert-informed strategy pending prospective validation.

Serial troponin monitoring has emerged as a valuable strategy for dynamic risk assessment. In a study of 455 patients undergoing serial high-sensitivity troponin I (hs-TnI) measurements, a follow-up hs-TnI level exceeding 8 ng/L was independently associated with increased all-cause mortality and cardiovascular events after multivariable adjustment (75). Similarly, among 135 patients treated with pembrolizumab, abnormally elevated baseline hs-TnI (>50 ng/L) predicted MACE with an eight-fold increased hazard, while follow-up elevations predicted MACE with a ten-fold increased hazard (76). The utility of baseline troponin measurement extends to identifying patients with pre-existing myocardial injury who may be at heightened vulnerability to ICI-associated cardiovascular toxicity (76). For CAR-T cell therapy, troponin elevations are also clinically relevant, as patients who experienced severe cardiovascular events had higher peak troponin levels following infusion, and the occurrence of these events was independently associated with increased overall mortality (77).

Natriuretic peptides, particularly NT-proBNP and BNP, have demonstrated prognostic value in cancer patients receiving ICI therapy (28, 78). In a cohort of 550 patients, baseline NT-proBNP levels were independently associated with an increased risk of cardiovascular hospitalization and death after adjustment for multiple confounders, including age, sex, renal function, and established cardiovascular risk factors (78). While natriuretic peptides are less specific for ICI-associated myocarditis compared to troponin, they provide complementary information regarding hemodynamic stress and heart failure risk, and their integration into routine surveillance protocols is recommended by expert consensus statements (28, 79). The ongoing CAVACI trial, a prospective multicenter study, incorporates both troponin and NT-proBNP measurements at fixed time points to evaluate their cumulative incidence and evolution during ICI therapy, which will provide prospective data to inform monitoring guidelines (79).

Beyond cardiac-specific biomarkers, non-cardiac biomarkers play important roles in the diagnosis and prognostication of ICI-associated myocarditis, particularly CK and its myocardial fraction (CK-MB) (67, 80). CK elevations frequently precede symptomatic presentation and are highly sensitive for ICI-associated myocarditis, especially given the frequent co-occurrence with myositis (67). In patients with esophageal cancer, elevated CK-MB and CK levels were identified as significant risk factors for asymptomatic ICI-associated myocarditis, and their combination with age and sex in a predictive model improved diagnostic capability with an area under the receiver operating characteristic curve of 0.808 (80). The cardiac troponin-to-absolute lymphocyte count (cTn/ALC) ratio has emerged as a novel composite biomarker that integrates myocardial injury with immune activation status (81). In a retrospective analysis of 46 patients with ICI-associated myocarditis, an elevated cTn/ALC ratio was associated with the development of severe myocarditis and emerged as an independent predictor for MACE with a hazard ratio of 5.64 (81). This ratio may capture the synergistic effect of myocardial damage and lymphopenia, the latter reflecting immune exhaustion or sequestration.

Inflammatory cytokines represent a mechanistically relevant class of biomarkers that provide insights into the underlying pathophysiology of ICI cardiovascular toxicity (60, 82, 83). IL-6 and TNF-α are the most commonly elevated cytokines in patients with ICI-associated myocarditis, with 74% and 68% of patients, respectively, showing elevations above normal limits in one cohort (83). However, the prognostic utility of these cytokines remains uncertain, as elevated peak IL-6 and TNF-α levels were not significantly associated with 90-day mortality or MACE in the same study (83). A pilot study utilizing semi-quantitative cytokine array analysis identified that baseline immune dysregulation may predispose to ICI-associated myocarditis, with 43 cytokines, including G-CSF, IL-6, and CXCL13, significantly elevated before ICI initiation in patients who subsequently developed myocarditis compared to controls (82). After ICI treatment, 23 cytokines remained significantly higher in the myocarditis group, suggesting that both baseline and dynamic cytokine profiling may aid in risk prediction (82). For CAR-T cell therapy, the cytokine storm characteristic of CRS is directly linked to cardiovascular toxicity, and peak IL-6, C-reactive protein, and ferritin levels are significantly higher in patients who experience severe cardiovascular events (77). These biomarkers may guide the timing and intensity of immunosuppressive therapy, particularly IL-6 inhibition with tocilizumab.

While exciting pathophysiological insights are being generated, it is vital to recognize that the supporting evidence for many novel markers is currently derived from very small cohorts, retrospective analyses, or early proof-of-concept investigations. Emerging investigational biomarkers with potential for future risk stratification include clonal hematopoiesis of indeterminate potential (CHIP), the cTn/ALC ratio, and novel proteomic signatures (84, 85). Until validated by large-scale prospective trials, these remain strictly investigational and should not yet be implemented in routine practice. In a prospective cardio-oncology cohort, CHIP status doubled the risk of developing ICI-associated myocarditis in a multivariate competing risk analysis, with a hazard ratio of 2.74, comparable to the risk associated with dual ICI use (84). This finding suggests that somatic mutations in hematopoietic stem cells may predispose to exaggerated inflammatory responses upon immune checkpoint blockade, offering a potential pre-treatment screening tool (84). Proteomic approaches have identified differential protein expression profiles that may distinguish ICI-associated myocarditis from acute myocardial infarction, which presents a diagnostic challenge given overlapping troponin elevations (85). Two plasma proteins, myomesin 3 (MYOM3) and galectin-1 (LGALS1), were identified as potential biomarkers linked to ICI-associated myocarditis onset in both animal models and expanded clinical cohorts, highlighting their promise for improving diagnostic specificity (85). Additionally, advanced imaging biomarkers such as fibroblast activation protein inhibitor (FAPI) positron emission tomography (PET) and somatostatin receptor-based PET have shown high sensitivity for detecting myocardial inflammation even in early stages or when CMR imaging is negative (86, 87). In a study of 11 patients with suspected ICI-associated myocarditis, all nine who underwent 68Ga-DOTATOC PET/CT demonstrated pathological myocardial uptake, while only 38% had positive findings on CMR imaging, suggesting that molecular imaging may complement serum biomarkers for early diagnosis (87).

The integration of multiple biomarkers into composite risk scores and clinical algorithms represents the most promising approach for clinical implementation (30, 67, 71, 72). A practical guide to risk factors and biomarkers for irAEs categorizes biomarkers into circulating blood counts, cytokines, autoantibodies, HLA genotypes, microRNA, gene expression profiling, and serum proteins, with neutrophil-lymphocyte ratio, eosinophil counts, and thyroid-stimulating hormone among the readily accessible markers (30). The proposed framework for future research emphasizes the need for multisite registries with centralized biobanking to allow head-to-head evaluation of different biomarker assays and clinical validation in ICI-associated myocarditis (70). Such efforts will be essential to establish standardized thresholds, define optimal monitoring intervals, and develop evidence-based algorithms that incorporate biomarkers into routine clinical care for patients receiving immunotherapy (28, 70).

## 5 Advanced cardiovascular imaging in detection and monitoring

Cardiovascular imaging has become indispensable in the detection, diagnosis, risk stratification, and monitoring of immunotherapy-associated cardiovascular toxicity. The clinical presentation of ICI-associated myocarditis is highly variable, ranging from asymptomatic biomarker elevations to fulminant heart failure and cardiogenic shock, making imaging a cornerstone of the diagnostic algorithm (88, 89). Multimodal imaging approaches, integrating echocardiography, CMR, and molecular imaging techniques, provide complementary information on cardiac structure, function, tissue characterization, and inflammation that cannot be obtained from biomarkers or electrocardiography alone (90, 91).

Echocardiography remains the most widely used first-line imaging modality due to its accessibility, lack of ionizing radiation, and ability to assess both left and right ventricular function. LVEF is the most commonly reported parameter, but its sensitivity for detecting early cardiovascular toxicity is limited. In a large retrospective cohort of 2,165 patients treated with ICIs, 28% of those who underwent echocardiography after ICI initiation had abnormal LVEF or GLS (29). This finding highlights the high prevalence of subclinical cardiac dysfunction that may be missed by LVEF assessment alone. GLS, derived from speckle-tracking echocardiography, has emerged as a more sensitive marker of early myocardial injury. In an observational cohort of 129 patients receiving ICI therapy, an early relative worsening of ≥10% in basal and mid LV longitudinal strain and ≥15% in GLS was associated with subsequent hs-TnI elevation, suggesting that strain imaging can identify patients at risk for myocardial injury before biomarker elevations occur (92). Importantly, 4 of 6 patients who developed clinically overt myocarditis had a relative reduction of ≥10% in basal LS, underscoring the potential of regional strain analysis for early detection (92). Right ventricular (RV) function, often overlooked in standard echocardiographic assessment, may also be affected early in the course of ICI therapy. Right ventricular and atrial strain abnormalities may also serve as early indicators of cardiovascular toxicity that precede changes in standard echocardiographic measures (93). However, echocardiography has important limitations, including operator dependence, suboptimal acoustic windows in some patients, and limited tissue characterization capabilities, which constrain its diagnostic accuracy for myocarditis (94).

CMR has emerged as the reference standard noninvasive imaging modality for the evaluation of suspected ICI-associated myocarditis due to its unique ability to characterize myocardial tissue through parametric mapping and late gadolinium enhancement (LGE) (88, 89, 95). CMR provides a comprehensive assessment of cardiac anatomy, biventricular function, myocardial edema, fibrosis, and inflammation in a single examination. The diagnostic performance of CMR in ICI-associated myocarditis has been evaluated in several studies, with variable results depending on the diagnostic criteria used and the clinical context. In a study comparing 33 patients with ICI-associated myocarditis to 21 patients scheduled to receive ICI therapy and 85 patients with viral myocarditis, patients with ICI-associated myocarditis showed significantly higher global native T1, extracellular volume fraction (ECV), and T2 z scores compared with the pre-ICI group, and LGE was present in 82% of ICI-associated myocarditis patients vs. only 10% of the pre-ICI group (96). However, the pattern of LGE in ICI-associated myocarditis appeared distinct from that of viral myocarditis: LGE was less frequent overall in ICI-associated myocarditis (82% vs. 100%) but was more likely to involve the septal segments (48% vs. 29%) and the midwall layer (33% vs. 2%) (96). Septal LGE was the only CMR parameter that independently predicted MACE at 1 year, even after adjustment for peak troponin, with an adjusted hazard ratio of 2.7 (96). This finding suggests that the location and pattern of LGE, rather than its mere presence, carry important prognostic information in ICI-associated myocarditis.

The sensitivity of CMR for diagnosing ICI-associated myocarditis in real-world clinical practice appears lower than that reported in highly selected research cohorts. Real-world observational studies indicate that the low sensitivity of common CMR measures for early inflammation, such as LGE and T2 mapping, necessitates a comprehensive multimodal diagnostic approach (97). The limitations of CMR are further highlighted by studies comparing it with molecular imaging techniques. In a prospective study of 49 patients undergoing ICI therapy with repeated CMR, there were no significant changes in T1 or T2 mapping values over time, despite a significant decline in LVEF and the occurrence of MACE in 31% of patients at 6 months (34). These data indicate that parametric mapping may not be sufficiently sensitive to detect the subtle or patchy myocardial inflammation that characterizes early ICI cardiovascular toxicity.

While echocardiography and CMR represent the validated clinical cornerstones for non-invasive assessment, the limitations of conventional CMR have prompted the investigation of alternative imaging approaches, particularly molecular imaging using targeted tracers. Although FAPI PET/CT and 68Ga-DOTATOC PET/CT represent promising frontiers, they must be strictly classified as emerging investigational tools. Given that current data are derived from highly selected, small-scale proof-of-concept cohorts, these modalities are not yet ready for routine clinical adoption and require robust prospective validation. FAPI PET/CT, which targets fibroblast activation protein expressed by activated fibroblasts in inflamed and remodeling tissues, has shown promise for detecting ICI-associated myocarditis. In a retrospective study comparing FAPI PET/CT scans of 26 patients who received ICIs, three patients with clinically suspected ICI-associated myocarditis showed cardiac enrichment of the tracer, with a median standardized uptake value (SUV) of 1.79, compared with 1.15 in patients without signs of cardiac adverse effects (86). The authors suggested that FAPI PET/CT may be used to identify affected patients at an early stage and, when integrated into cancer staging diagnostics, could contribute to cardiac risk stratification alongside biomarkers, ECG, and echocardiography (86). Another molecular imaging approach uses 68Ga-DOTATOC PET/CT, which targets somatostatin receptors expressed on activated macrophages and other inflammatory cells. In a small proof-of-concept case series of 11 patients with clinically suspected ICI-associated myocarditis, all 9 patients who underwent 68Ga-DOTATOC PET/CT showed pathological myocardial uptake suggestive of myocarditis, with a myocardium-to-background ratio of peak SUV to mean intracavitary LV SUV of 3.2 ± 0.8 (87). In contrast, only 3 of 8 patients (38%) who underwent CMR had findings evocative of myocarditis. Notably, 5 of 6 patients who presented with concomitant myositis showed pathological tracer uptake in skeletal muscles on whole-body imaging, suggesting an additional advantage of this method for assessing the full extent of irAEs (87). The high sensitivity of 68Ga-DOTATOC PET/CT was particularly evident in patients with elevated cTnI but negative CMR findings, indicating that molecular imaging may detect myocardial inflammation at an earlier stage than CMR (87). These findings highlight the potential complementary roles of CMR and molecular imaging: CMR provides detailed structural and functional information with high specificity for established myocardial injury, while molecular imaging may offer higher sensitivity for detecting early or low-grade inflammation.

The integration of advanced imaging into clinical care pathways is evolving, with growing recognition that a single imaging modality may be insufficient for the comprehensive assessment of ICI cardiovascular toxicity. Multimodal imaging strategies that combine echocardiography, CMR, and molecular imaging, tailored to the clinical presentation and the specific questions being addressed, are increasingly recommended (88, 90, 91). For example, echocardiography with strain imaging may serve as an initial screening tool for subclinical dysfunction, followed by CMR for tissue characterization in patients with suspected myocarditis, and expert-informed proposals suggest considering molecular imaging for cases where CMR is negative but clinical suspicion remains high (87, 89), though this escalation pathway requires further robust clinical validation. The timing of imaging is also critical: baseline imaging prior to ICI initiation can identify pre-existing cardiac abnormalities that may predispose to toxicity, while serial imaging during therapy can detect early changes that precede clinical events. In a prospective cohort study of 57 patients undergoing ICI therapy, baseline and follow-up CMR at approximately 2 months revealed a significant LVEF reduction from 60.1% to 58.1%, with more than 3% LVEF loss in 32% of patients (98). These patients also showed significantly higher T2 values, a putative sign of cardiac edema, suggesting that subclinical myocardial inflammation may be more common than previously recognized (98). The loss of cardiac function did not differ among patients with different tumor types, therapy regimens, or history of cardiac disease, indicating that routine imaging surveillance may be warranted across all ICI-treated populations (98).

Despite the progress in cardiovascular imaging for ICI cardiovascular toxicity, several challenges remain. The diagnostic criteria for ICI-associated myocarditis are not uniformly defined, and the performance characteristics of different imaging modalities vary depending on the reference standard used. The Lake Louise Criteria, originally developed for viral myocarditis, have been applied to ICI-associated myocarditis but may have suboptimal sensitivity in this population due to the different pathophysiological mechanisms and the often patchy nature of myocardial inflammation (89, 96, 97). The presence of pre-existing cardiovascular disease, which is common in cancer patients, can confound the interpretation of imaging findings, as LGE from prior myocardial infarction or other cardiomyopathies may be indistinguishable from ICI-associated injury (97). Furthermore, the availability of advanced imaging techniques, particularly CMR and PET/CT, is limited in many clinical settings, and the optimal surveillance intervals and imaging protocols have not been established through prospective randomized trials (90, 91). The development of standardized imaging protocols and reporting criteria specific to ICI cardiovascular toxicity, as well as the integration of imaging findings with clinical and biomarker data into validated risk stratification models, represents a critical unmet need (Table 2).

**Table 2:** Multimodal diagnostic toolsets and imaging indicators for immunotherapy-induced cardiovascular toxicity.

Modality	Specific Indicator	Primary Clinical Application	Advantages	Limitations
Cardiac Biomarkers	hs-cTnT	Early screening, diagnosis, and prognostication	Demonstrates superior diagnostic sensitivity compared to troponin I; magnitude of elevation correlates strongly with disease severity	Clinical interpretation of isolated troponin elevations in asymptomatic patients remains an area of active investigation
(Biomarkers)	NT-proBNP/BNP	Assessment of hemodynamic stress and heart failure risk	Provides complementary prognostic value and reflects subclinical cardiovascular stress	Less specific for ICI-associated myocarditis compared to troponin
Echocardiography	GLS (Speckle-tracking)	Detection of early or subclinical myocardial dysfunction	Non-invasive and sensitive for identifying patients at risk before biomarker elevations occur	Operator-dependent and constrained by limited tissue characterization capabilities
Advanced Imaging (CMR)	T1/T2 Mapping, LGE	Reference standard noninvasive modality for suspected ICI-associated myocarditis	Comprehensive assessment of biventricular function, edema, and fibrosis; septal LGE independently predicts MACE	Low sensitivity of common measures for early inflammation; confounded by LGE from prior myocardial infarction
Molecular Imaging (PET)	FAPI & 68Ga-DOTATOC	Detection of early macrophage and fibroblast activation	High sensitivity for detecting low-grade myocardial inflammation, even when CMR findings are negative	Remains an emerging investigational tool not yet ready for routine clinical adoption; supporting evidence is currently limited to very small, retrospective proof-of-concept cohorts; limited availability; lacks established standardized imaging protocols specific to ICI cardiovascular toxicity

## 6 Risk factors, prevention strategies, and predictive models

The identification of patients at heightened risk for ICI-associated cardiovascular toxicity is a cornerstone of preventive cardio-oncology, yet this field remains characterized by significant knowledge gaps. The relationship between sex and ICI cardiovascular toxicity appears to be nuanced and agent-dependent. A retrospective review of 538 patients revealed that female sex was a significant risk factor for cardiotoxic events, with an incidence rate ratio of 3.34 (99). However, a broader review of the literature suggests that the sex effect may be modified by the specific ICI class, with women potentially at higher risk with CTLA-4 inhibitors and men with PD-1/PD-L1 agents (30). Preclinical work has provided mechanistic insight into this sexual dimorphism, demonstrating that ICI treatment reduces serum estradiol concentrations, leading to downregulation of MANF and HSPA5 in the heart, a pathway that preferentially affects female mice and can be attenuated by estrogen receptor β agonists (61). Age also plays a role, with younger age (<60 years) associated with a higher overall risk of irAEs, while older age has been specifically linked to a higher risk of cardiac fatality in the adjuvant setting (30, 100).

Pre-existing cardiovascular disease and its risk factors constitute a critical domain of risk. A history of coronary artery disease, heart failure, myocardial infarction, and hypertension has been associated with an elevated risk of organ-specific cardiac irAEs (30). In a study of 378 patients, a history of arrhythmia or ischemic heart disease was significantly associated with cardiac events on multivariable analysis (101). Furthermore, pre-existing atherosclerotic cardiovascular disease (ASCVD) was independently associated with subsequent ASCVD events during ICI therapy (HR 4.00), with events occurring even after ICI cessation, underscoring the need for sustained risk factor management (102). The metabolic syndrome, conceptualized within the cardiovascular-kidney-metabolic framework, compounds this risk; in patients with hepatocellular carcinoma (HCC), the presence of both arterial hypertension and type 2 diabetes mellitus amplified the risk of MACE and treatment discontinuation (103). The importance of pre-existing cardiac injury as a prerequisite for severe myocarditis has been demonstrated in a hypertension mouse model, where ICI combination therapy induced lethal myocarditis with T cell infiltration and cardiac autoantibodies only in hypertensive mice, while control mice remained unaffected (62).

Tumor-specific factors also contribute to risk. The presence of active thymoma emerged as the strongest predictor of adverse outcomes in a large multicenter registry of 748 patients with ICI-associated myocarditis, conferring a hazard ratio of 3.6 for the composite endpoint of severe arrhythmia, heart failure, respiratory muscle failure, or death (71, 72). This association may relate to the unique immune microenvironment of thymic tumors and their propensity for paraneoplastic autoimmune phenomena. Additionally, the Khorana score, a clinical tool for predicting venous thromboembolism, was significantly associated with thromboembolic events during ICI therapy (HR 2.40), suggesting that cancer-related hypercoagulability interacts with ICI-associated immune activation (104).

Treatment-related factors are among the most modifiable and well-characterized risks. Combination ICI therapy (e.g., anti-PD-1 plus anti-CTLA-4) is a predominant risk factor for myocarditis, with dual ICI use conferring a risk similar in magnitude to clonal hematopoiesis in one prospective study (31, 84). The addition of ICI to other cardiotoxic anticancer therapies, such as tyrosine kinase inhibitors, radiation, or chemotherapy, further escalates risk (31). In the JAVELIN Renal 101 trial, which prospectively monitored patients receiving avelumab plus axitinib vs. sunitinib, MACE occurred in 7.1% of the combination arm, and high baseline troponin T levels identified a subgroup at 3.31-fold higher relative risk (105). This finding underscores the potential value of baseline cardiac biomarkers for risk stratification, even in the absence of formalized screening protocols.

Emerging evidence points to novel biomarkers that may refine risk prediction beyond traditional factors. CHIP, an age-related phenomenon involving somatic mutations in hematopoietic stem cells, was shown in a prospective study to double the risk of ICI-associated myocarditis (HR 2.74), independent of dual ICI use and other covariates (84). This finding aligns with the known role of CHIP in promoting T cell activation and systemic inflammation, and it positions CHIP as a potentially powerful tool for pre-treatment risk stratification. Other readily accessible biomarkers include the neutrophil-lymphocyte ratio, eosinophil counts, and thyroid-stimulating hormone levels, which have been associated with irAE risk (30). For asymptomatic myocarditis specifically in patients with esophageal cancer, a combination of age, sex, and CK isoenzyme levels demonstrated improved predictive value (AUC 0.808) compared to individual parameters (80).

Several prognostic risk scores have been developed to synthesize these risk factors into clinically actionable tools. The most extensively validated is a point-based risk score derived from the international multicenter registry of 748 patients with ICI-associated myocarditis (71, 72). This score incorporates five independent predictors: active thymoma, presence of cardiomuscular symptoms, low QRS voltage on presenting electrocardiogram, LVEF < 50%, and incremental troponin elevation. Notably, this risk score was used prospectively to identify low-risk patients who were safely managed without immunosuppression, resulting in no cardiomyotoxic events, suggesting its potential to guide therapeutic intensity (71). For broader cardiovascular risk assessment in the ICI population, the CARDIOSOR scale, which incorporates hypertension and diabetes, effectively stratified patients with HCC into high-risk groups correlating with increased MACE rates (103). Furthermore, the integration of Artificial Intelligence (AI) and machine learning is poised to transform precision risk stratification in cardio-oncology. Beyond traditional clinical risk scores, AI algorithms are increasingly being trained to perform automated, high-throughput analysis of standard 12-lead ECGs and echocardiographic GLS images (106). These advanced machine learning models possess the capability to detect subtle, subclinical patterns of myocardial deformation and repolarization abnormalities that may elude routine visual assessment (107). By identifying these covert signatures of early myocardial stress, AI models can significantly enhance real-time predictive capacity in daily clinical practice, allowing for preemptive cardioprotective interventions before overt clinical toxicity manifests (108). Although large-scale validation of these AI tools specifically within ICI-treated cohorts is still maturing, they represent a critical frontier in personalized surveillance.

While the identification of high-risk patients is advancing, it is crucial to emphasize that there are currently no established prophylactic standards specifically validated for immunotherapy-induced cardiovascular toxicity. Prevention strategies discussed herein remain largely hypothesis-generating and are extrapolated from observational data or broader cardiovascular guidelines. For instance, in observational cohorts, influenza vaccination has been hypothesized to be a potentially protective measure, with vaccinated patients showing lower rates of myocarditis (109). Pharmacological prevention with statins has also garnered attention as a promising hypothesis (110). While a propensity score-matched cohort study found that statin use was associated with a 13% relative risk reduction in MACE (111), this data is retrospective and requires prospective confirmation before clinical adoption. Similarly, the management of the cardiovascular-kidney-metabolic syndrome through sodium-glucose cotransporter-2 (SGLT2) inhibitors, proprotein convertase subtilisin/kexin type 9 (PCSK9) inhibitors, and lifestyle interventions represents an extrapolated conceptual framework from broader cardio-oncology paradigms, rather than an evidence-based immunoprophylaxis recommendation (112). The integration of these risk factors, biomarkers, and predictive models into a comprehensive pre-treatment assessment, coupled with evidence-based preventive strategies, holds the potential to reduce the burden of ICI-associated cardiovascular toxicity while preserving the oncologic benefits of immunotherapy (Table 3).

**Table 3:** Risk stratification domains and clinical prophylactic implications.

Risk Domain	Specific Risk Factor	Risk Characteristics and Impact	Clinical Monitoring and Preventive Implications
Patient Demographics & Comorbidities	Pre-existing CVD, cardiovascular-kidney-metabolic Syndrome	Associated with an elevated risk of organ-specific cardiac irAEs; pre-existing injury lowers the threshold for immune-mediated attack	Optimization of cardiovascular-kidney-metabolic health; statin use is associated with a relative risk reduction in MACE
(Demographics & Comorbidities)	Female Sex	Female patients appear to be preferentially affected; preclinical data suggest this is potentially due to ICI-associated reduction in estradiol and downregulation of cardioprotective MANF and HSPA5 pathways	Vigilant monitoring in female patients, with some evidence suggesting higher risk with CTLA-4 inhibitors
Tumor-Specific Factors	Active Thymoma	Emerged as the strongest predictor of adverse outcomes (HR: 3.6 for composite endpoints)	High index of suspicion and rigorous baseline and serial cardiac evaluation
Treatment Regimen	Dual ICI Therapy (anti-CTLA-4 + anti-PD-1/PD-L1)	The most consistently reported potent risk factor, demonstrating a 3.07-fold higher risk compared to single ICI use	Heightened clinical vigilance and serial biomarker surveillance, particularly during the initial weeks of therapy
Emerging Biomarkers	CHIP	Doubles the risk of developing ICI-associated myocarditis (HR: 2.74), independent of dual ICI use	Represents a potential pre-treatment screening tool to identify heightened vulnerability to inflammatory responses

## 7 Current management approaches and emerging therapies

### 7.1 Risk-Based approach and Pre-treatment assessment

The management of immunotherapy-associated cardiovascular toxicity, particularly ICI-associated myocarditis, has evolved from anecdotal experience to a structured, severity-based framework guided by international consensus statements and position papers (12, 39, 113) (Figure 2). As illustrated in our decision algorithm (Figure 2), we propose a comprehensive clinical workflow stratified into four sequential phases: pre-treatment risk stratification (Step 1), routine non-invasive surveillance (Step 2), advanced multimodal diagnosis (Step 3), and stratified management (Step 4). However, it is crucial to emphasize that this framework represents a proposed, expert-informed conceptual pathway. Several components within this algorithm—particularly routine serial biomarker surveillance, molecular PET imaging, and risk-score-directed treatment intensity—are not yet supported by high-level prospective evidence and may not reflect universally adopted standards of care across all institutions. The cornerstone of this framework is the immediate and coordinated multidisciplinary assessment involving cardiologists, oncologists, and, when available, intensivists, as the clinical trajectory of ICI-associated myocarditis can be unpredictable and rapidly progressive (12, 114, 115). The initial step in management is the accurate grading of severity, which directly informs the intensity of intervention.

**Figure 2:**
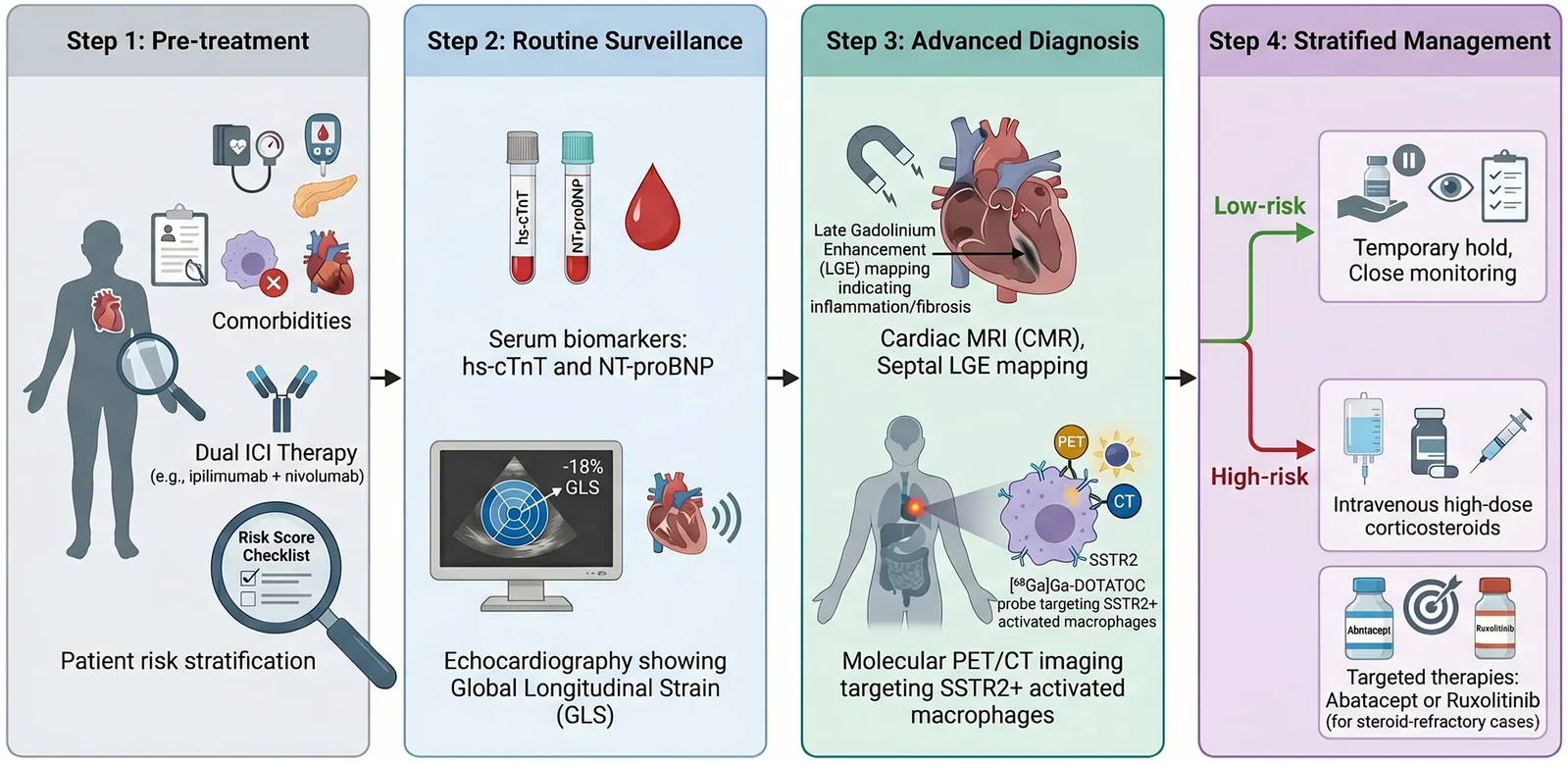
Proposed stepwise clinical workflow for the surveillance and management of immunotherapy-associated cardiovascular toxicity.

### 7.2 Management of the asymptomatic patient

For asymptomatic patients with isolated troponin elevation (often termed subclinical myocarditis), current guidelines recommend close monitoring with serial biomarkers and electrocardiograms, temporary holding of ICI therapy, and consideration of low-dose corticosteroids if troponin levels continue to rise or if other concerning features emerge (12, 116). In a single-center experience, 25% of patients presented with this subclinical form, and the majority were managed with systemic corticosteroids and ICI interruption, with good outcomes (116). This approach aligns with the principle that early detection and intervention can prevent progression to more severe forms (117, 118).

This multidisciplinary algorithmic approach is stratified into four sequential phases. It is important to note that this represents a conceptual framework based on current expert consensus and emerging data, rather than a universally validated standard-of-care. Step 1 (Pre-treatment) emphasizes baseline patient risk stratification, focusing on pre-existing comorbidities and the implementation of high-risk regimens such as dual immune checkpoint inhibitor (ICI) therapy (e.g., ipilimumab and nivolumab) utilizing a standardized risk score checklist. Step 2 (Routine Surveillance) outlines non-invasive monitoring strategies, combining the assessment of serial serum biomarkers (hs-cTnT and NT-proBNP) with early functional evaluation via echocardiography-derived GLS. Step 3 (Advanced Diagnosis) is indicated for suspected cases, utilizing cardiac MRI (CMR) with late gadolinium enhancement (LGE) mapping to evaluate septal inflammation and fibrosis, alongside molecular PET/CT imaging with a 68Ga-DOTATOC probe to precisely target SSTR2+ activated macrophages. Finally, Step 4 (Stratified Management) guides therapeutic interventions based on clinical severity: low-risk patients undergo temporary ICI suspension and close monitoring, whereas high-risk patients require immediate intravenous high-dose corticosteroids, with targeted therapies such as abatacept or ruxolitinib strategically reserved as escalation strategies for steroid-refractory cases. (Created with BioRender.com).

### 7.3 Emergency management for symptomatic, fulminant, and refractory cases

For patients with symptomatic ICI-associated myocarditis, defined by the presence of cardiac symptoms, electrocardiographic abnormalities, or hemodynamic instability, high-dose corticosteroids remain the first-line and most critical intervention (39, 119–121). The recommended regimen typically involves intravenous methylprednisolone at doses of 500 mg to 1,000 mg daily for three to five days, followed by a gradual oral prednisone taper over four to six weeks (12, 115, 121). The biological rationale for high-dose steroids is the rapid suppression of the aberrant T-cell-mediated immune response targeting cardiomyocytes (122, 123). The importance of early and aggressive corticosteroid administration has been underscored by a neural net modeling study of 23 patients with ICI-associated myocarditis, which identified early steroid treatment as a major determinant of survival for cases of grade 3 or 4 severity (124). A registry-based analysis from the SERIO cohort, which included centers with established troponin monitoring protocols and consequent treatment pathways, reported a mortality rate of only 11.3% for cardiac irAEs, substantially lower than the historically cited 40%–50% (117). This finding suggests that a systematic approach to early detection and prompt initiation of corticosteroids can significantly improve outcomes (117, 124).

Despite the efficacy of corticosteroids in the majority of cases, a substantial proportion of patients exhibit steroid-refractory or steroid-resistant disease, defined by persistent or worsening cardiac biomarkers, arrhythmias, or hemodynamic compromise despite high-dose steroid therapy (121, 125). The management of these high-risk patients is one of the most challenging aspects of ICI cardiovascular toxicity. When navigating this challenge, it is imperative for clinicians to clearly distinguish established standards from investigational approaches. High-dose corticosteroids remain the universally established standard first-line therapy. In contrast, all subsequent steroid-sparing or escalation agents—including abatacept, ruxolitinib, intravenous immunoglobulin (IVIG), mycophenolate mofetil, antithymocyte globulin (ATG), and infliximab—remain based on limited evidence derived primarily from observational data, expert consensus, and case reports. Consequently, the application of these second-line therapies represents a rapidly evolving area of clinical practice rather than universally validated treatment pathways. A landmark case-control study comparing patients who required intensified immunosuppressive therapy (IIST) to those who did not found that the need for IIST was more common in patients who developed myocarditis very early after ICI initiation (median 18 days vs. 60 days), and in those presenting with hemodynamic or electrical instability, or concurrent neuromuscular adverse events such as myositis or myasthenia gravis (126). Patients requiring IIST had a significantly higher all-cause mortality (50% vs. 21%), highlighting the gravity of this clinical scenario (126). The choice of second-line agent remains a subject of active investigation, with no single agent demonstrating clear superiority in randomized trials (121, 125). Based primarily on expert consensus and retrospective case series, current guidelines recommend several options, including mycophenolate mofetil, intravenous immunoglobulin, antithymocyte globulin, and TNF-α inhibitors such as infliximab (119, 120, 127, 128). However, the use of infliximab has been associated with a concerning signal of increased cardiovascular death. In the same case-control study, patients who received infliximab were more likely to die from cardiovascular causes compared to those who received other IIST agents (OR 12.0; 95% CI 2.1–67.1) (126). This observation has led to caution regarding the routine use of TNF-α inhibitors in ICI-associated myocarditis, particularly given that TNF-α inhibitors are contraindicated in patients with moderate-to-severe heart failure from other causes (125, 126).

Emerging targeted therapies represent a paradigm shift in the management of steroid-refractory ICI-associated myocarditis, moving beyond broad immunosuppression toward pathway-specific immunomodulation. Abatacept, a CTLA-4 fusion protein, has garnered significant attention as a potentially targeted therapy (121, 129). The rationale for abatacept is its ability to inhibit T-cell co-stimulation by binding to CD80/CD86 on antigen-presenting cells, thereby counteracting the unchecked T-cell activation induced by ICI therapy (121, 129). Abatacept is currently the only drug under investigation in a randomized clinical trial for ICI-associated myocarditis, representing a critical step toward evidence-based management (121). Case reports have described successful use of abatacept in fulminant, steroid-refractory myocarditis, often in combination with other agents (129). Another promising agent is ruxolitinib, a Janus kinase (JAK) inhibitor that impairs immune activation by suppressing cytokine sensing and production, as well as T-cell activation (129, 130). In a reported case, following the initiation of off-label ruxolitinib, the patient experienced sustained clinical improvement, return to sinus rhythm, and recovery of biventricular function (130). These cases, while anecdotal, highlight the potential of JAK inhibition as a targeted strategy to interrupt the inflammatory cytokine cascade driving myocardial injury (58, 129, 130). While head-to-head comparative data detailing the relative efficacy of these escalation regimens are currently lacking, emerging clinical consensus suggests tailoring the choice of biological agent to the specific patient phenotype. Abatacept is increasingly favored as the first-line escalation therapy for isolated, severe steroid-refractory myocarditis due to its direct and potent inhibition of T-cell co-stimulation (125). Conversely, ruxolitinib or alternative targeted therapies may be preferred in cases where myocarditis presents alongside massive systemic inflammation, cytokine release features, or distinct overlap syndromes (131). The ongoing randomized ATRIUM trial (NCT05335928), which formally evaluates abatacept vs. placebo in ICI-associated myocarditis, is highly anticipated to provide the definitive, evidence-based data required to standardize these complex escalation algorithms (121).

While acute myocarditis necessitates intensive immunosuppression, the broader spectrum of immunotherapy-induced cardiovascular toxicities requires tailored, disease-specific approaches. Pericarditis and Pericardial Effusion: Isolated cases are managed primarily with nonsteroidal anti-inflammatory drugs (NSAIDs) or colchicine. However, these agents should be used cautiously, and concurrent systemic corticosteroids are indicated if pericarditis overlaps with myocarditis or severe systemic irAEs (12, 115, 132). Arrhythmias and Conduction Disorders: These complications demand aggressive continuous monitoring. Severe bradyarrhythmias or complete heart block frequently require temporary or permanent pacemaker implantation, while tachyarrhythmias are managed with standard antiarrhythmic protocols alongside immediate ICI interruption (12, 116, 126, 133). Non-inflammatory Heart Failure and Takotsubo Syndrome: These entities are generally managed according to standard guideline-directed medical therapy (GDMT) for heart failure (115, 134). In cases of hemodynamic instability or cardiogenic shock, temporary mechanical circulatory support with devices such as percutaneous microaxial flow pumps may serve as a critical bridge to recovery (129). Vasculitis and Accelerated Atherosclerosis: Immune-related vasculitis typically responds favorably to high-dose glucocorticoids. Conversely, mitigating the long-term risk of accelerated atherosclerosis demands stringent cardiometabolic optimization and aggressive lipid-lowering strategies rather than acute immunosuppression.

### 7.4 ICI rechallenge and long-term management

The decision to rechallenge with ICI therapy after an episode of myocarditis is one of the most complex clinical decisions in cardio-oncology (12, 120, 135). Current consensus suggests that ICI rechallenge may be considered in select cases, particularly for patients who had mild or moderate myocarditis that fully resolved with treatment, and for whom no alternative effective cancer therapy exists (12, 115, 120). However, the risk of recurrence is substantial, and severe or fatal recurrent myocarditis has been reported (120). A multidisciplinary tumor board involving cardiology and oncology is essential for shared decision-making (12, 114). For patients who had severe or fulminant myocarditis, permanent discontinuation of ICIs is generally recommended (12, 115). If rechallenge is pursued, it should be done with close monitoring, including frequent troponin and electrocardiogram assessments, and consideration of using a single-agent ICI rather than combination therapy, as combination regimens carry a higher risk of myocarditis (12, 39, 126). The optimal timing for rechallenge is not established, but most experts recommend waiting until the myocarditis has completely resolved and corticosteroids have been tapered to a low dose or discontinued (115). In a systematic review of Takotsubo cardiomyopathy in ICI-treated patients, immunotherapy was reintroduced in 50% of cases, suggesting that this specific manifestation may have a more favorable risk profile for rechallenge (136).

Long-term management extends beyond the acute episode. Patients who have experienced ICI-associated myocarditis require ongoing cardiovascular surveillance, as the long-term consequences of myocardial inflammation are not fully understood (115, 135). Serial echocardiography with GLS assessment and cardiac biomarker monitoring are recommended to detect late-onset left ventricular dysfunction or heart failure (121, 135). The role of CMR imaging in long-term follow-up is emerging, as it can detect residual myocardial fibrosis even in patients with recovered LVEF (12, 115). Additionally, the management of cardiovascular risk factors, including hypertension, diabetes, and hyperlipidemia, remains a priority, as ICI therapy may accelerate atherosclerosis and increase the risk of acute coronary syndromes (12, 135). Structuring this long-term follow-up requires a proactive and systematic multidisciplinary approach. Following the conclusion of immunotherapy, or upon recovery from an acute cardiovascular irAE, current literature proposes implementing cardiovascular screening at standardized intervals—typically at 3, 6, and 12 months post-treatment, and annually thereafter (137). This structured surveillance should mandate a comprehensive clinical assessment, aggressive lipid profile monitoring, standard ECG, and serial echocardiography. Such vigilance is particularly crucial for identifying the subclinical progression of accelerated atherosclerosis, thereby facilitating the timely initiation of lipid-lowering therapies (e.g., statins or PCSK9 inhibitors) and strict cardiometabolic optimization to mitigate long-term ischemic risks (138, 139). The integration of cardioprotective medications such as statins and sodium-glucose cotransporter-2 inhibitors may offer dual benefits for both cardiovascular risk reduction and potential immunomodulation (110–112). As the field of cardio-oncology continues to evolve, the development of prospective registries and randomized controlled trials, such as the ongoing abatacept trial, will be critical to refine management algorithms and establish evidence-based therapies for this rare but devastating complication of modern cancer immunotherapy (58, 113, 121).

## 8 Special populations, overlap syndromes, and long-term outcomes

The clinical landscape of immunotherapy-associated cardiovascular toxicity is further complicated by the emergence of overlap syndromes, the influence of pre-existing comorbidities, and the need for long-term surveillance in cancer survivors. Overlap syndromes, particularly the concurrent presentation of myocarditis with myositis and myasthenia gravis (MG), represent a distinct and particularly high-risk phenotype. This “terrible triad” of irAEs has been increasingly recognized in patients receiving combination ICI therapy, especially with ipilimumab and nivolumab (140–142). The simultaneous occurrence of these conditions suggests a shared, severe pathophysiological mechanism against striated muscle antigens (143). Clinically, these patients often present with a rapidly progressive course, including severe bulbar weakness, respiratory failure, and life-threatening cardiac arrhythmias or heart failure, necessitating prompt and aggressive immunosuppression (140, 142, 144). The diagnostic challenge is considerable, as symptoms of myositis (e.g., proximal muscle weakness, elevated CK) can overlap with and mask the cardiac manifestations of myocarditis, delaying critical cardiac evaluation (145, 146). Studies have shown that in patients with ICI-associated myositis overlapping with myocarditis, troponin levels often peak later than CK levels and can remain elevated for several months after immunosuppressive therapy, indicating a more protracted and potentially smoldering cardiac injury (146). This temporal discordance between biomarkers underscores the need for prolonged cardiac monitoring in this population, even after initial clinical improvement (146). The presence of MG further complicates management, as the use of cholinesterase inhibitors may be less effective and the risk of myasthenic crisis is high (143, 144). Cases of refractory overlap syndrome have been successfully managed with rituximab, highlighting the potential role of B-cell depletion in severe, treatment-resistant cases (140).

Beyond the overlap with neuromuscular disease, ICI therapy has been associated with a spectrum of other cardiovascular overlap conditions. Takotsubo syndrome (TTS), or stress cardiomyopathy, can occur concurrently with ICI-associated myocarditis, presenting a diagnostic dilemma (147, 148). This overlap highlights the importance of comprehensive imaging, as the initial echocardiographic pattern of TTS can mask an underlying myocarditis, which may require distinct treatment with immunosuppression (148). Similarly, ICI-associated pericarditis or perimyocarditis (involvement of both the pericardium and myocardium) has been documented, presenting with chest pain, ST-segment elevation, and pericardial effusion, and often responding to corticosteroids and colchicine (149). Furthermore, ICI therapy has been linked to a variety of vasculitic syndromes, ranging from large-vessel vasculitis (LVV) mimicking giant cell arteritis to small-vessel vasculitis presenting as acral ischemia (150–152). A European multicenter study of ICI-associated LVV reported a median onset of just 3 months after therapy initiation, with a high rate of sustained remission (74%) after glucocorticoid treatment, though one-third of patients died during follow-up, predominantly from cancer progression (152). Cases of renal vasculitis, including medium-vessel involvement, have also been identified on pathological examination of nephrectomy specimens, suggesting this complication may be underrecognized (153). These diverse presentations underscore the systemic nature of immune dysregulation induced by ICIs.

The long-term cardiovascular outcomes in patients who survive acute ICI-associated cardiovascular toxicity remain an area of active investigation. While many patients recover left ventricular function with prompt immunosuppression, evidence suggests that a significant proportion may develop chronic, smoldering myocardial injury or fibrosis. The concept of “smoldering myocarditis” was first described in an isolated case report of a patient who, despite initial clinical improvement, had a recurrence of troponin elevation upon steroid taper, with endomyocardial biopsy showing ongoing lymphocytic inflammation and collagen fibrosis (154). This pattern of subclinical, persistent myocardial damage may be more common than previously appreciated, particularly in patients treated with combination ICI therapy (155). Long-term follow-up studies are scarce, but case series indicate that troponin levels can remain elevated for months after the acute event, and CMR may detect residual myocardial fibrosis or edema even in patients with normalized ejection fraction (146). The clinical significance of these findings is unclear, but they raise concerns about an increased risk of late-onset heart failure, arrhythmias, and sudden cardiac death. For instance, patients who have recovered from ICI-associated myocarditis may later develop atrial fibrillation or require permanent pacemaker placement for persistent conduction abnormalities, such as complete heart block (142, 156). The risk of accelerated atherosclerosis is another critical long-term concern. Preclinical models suggest that ICI therapy can promote plaque development and progression through T-cell dysregulation and proatherogenic cytokine production (157, 158). Furthermore, a retrospective study of HCC patients treated with atezolizumab-bevacizumab found that MACE, including myocarditis, occurred in 7.3% of patients and were independently associated with worse overall survival (103). These findings emphasize the need for vigilant, long-term cardiovascular risk factor management in all ICI-treated patients, including aggressive control of hypertension, diabetes, and hyperlipidemia (158).

The management of ICI-associated cardiovascular toxicity in special populations, such as those with pre-existing cardiovascular disease or those requiring subsequent cardiotoxic therapies, presents unique challenges. Patients with pre-existing conditions like hypertension and type 2 diabetes mellitus are at a significantly higher risk for developing MACE during ICI therapy, as demonstrated by the CARDIOSOR scale in HCC patients (103). This underscores the importance of baseline cardiovascular risk assessment and optimization before initiating immunotherapy. The safety of subsequent cancer therapies after ICI-associated myocarditis is also a critical question. Another complex scenario involves patients with cardiac metastases or pre-existing cardiomyopathy who are candidates for CAR-T therapy. A case series of lymphoma patients with cardiac involvement or cardiomyopathy managed with CAR-T therapy highlights the potential for life-threatening complications, including ventricular rupture and cardiac tamponade, and underscores the need for individualized risk-benefit assessment (159). Finally, the differential diagnosis of cardiac injury in ICI-treated patients must always consider non-ICI causes, such as epicardial metastases causing coronary artery compression or amyloidosis presenting with persistently elevated troponin (160, 161).

## 9 Conclusion and future perspectives

Over the past decade, immunotherapy-associated cardiovascular toxicity has evolved from sporadic case reports into a defined clinical entity with established diagnostic criteria and management algorithms. Significant preclinical advances have elucidated key pathophysiological drivers, including the role of cardiac myosin-specific autoimmune T cells, interleukin-17A, and the suppression of estrogen-dependent protective factors (MANF and HSPA5). These mechanistic insights are paving the way for targeted interventions, such as abatacept and ruxolitinib, for severe ICI-associated myocarditis. Concurrently, the development of robust, validated risk scores—incorporating clinical parameters like thymoma status and highly sensitive cardiac troponin T—has substantially improved early detection and risk stratification.

Clinical management has increasingly shifted toward a nuanced, severity-based approach. Stringent monitoring and structured interventions, which distinguish between asymptomatic biomarker elevations and symptomatic disease requiring intense immunosuppression, have successfully reduced historically high mortality rates. Furthermore, the cardiovascular toxicity spectrum is now recognized to extend well beyond acute myocarditis, encompassing long-term risks such as accelerated atherosclerosis. For CAR-T therapies, cardiovascular complications remain primarily driven by CRS, where targeted treatment with anti-IL-6 receptor antibodies like tocilizumab remains the cornerstone of care.

It is important to explicitly acknowledge the inherent limitations of this narrative review and the prevailing evidence base it synthesizes. First, the relative rarity of immunotherapy-associated cardiovascular toxicities, combined with heterogeneity in myocarditis definitions and constantly evolving diagnostic criteria, complicates the synthesis of uniform incidence rates and clinical outcomes. Second, much of the current literature heavily relies on retrospective cohorts and referral-center registries, which are intrinsically susceptible to selection bias. Furthermore, there is a probable publication bias that disproportionately favors the reporting of severe or fulminant cases over milder or subclinical presentations. Finally, while ICI-associated cardiovascular toxicity is increasingly characterized, robust cardiovascular datasets specific to CAR-T cell therapies remain notably limited. Consequently, the narrative conclusions and proposed algorithms presented herein should be interpreted with these methodological caveats in mind.

Despite these milestones, critical knowledge gaps necessitate future investigation. The long-term cardiovascular sequelae of ICIs, the optimal duration for surveillance, and the efficacy of preventive strategies (e.g., statin therapy) remain poorly defined. The safety of sequential cardiotoxic therapies following ICI-associated myocarditis also requires clarification. Future research must prioritize the rigorous prospective validation of emerging investigational tools (such as CHIP, proteomic signatures, and molecular imaging) to safely transition them from proof-of-concept studies to personalized predictive models. Additionally, exploring cardiometabolic therapies (e.g., SGLT2 and PCSK9 inhibitors) within the cardiovascular-kidney-metabolic syndrome framework, alongside sex-specific prophylactic approaches, represents a promising frontier. Addressing these challenges will require sustained, multidisciplinary collaboration and the expansion of international registries to establish comprehensive, evidence-based guidelines for this evolving patient population.
